# Omission of sentinel lymph node biopsy in patients with clinically axillary lymph node-negative early breast cancer (OMSLNB): protocol for a prospective, non-inferiority, single-arm, phase II clinical trial in China

**DOI:** 10.1136/bmjopen-2024-087700

**Published:** 2024-09-10

**Authors:** Xuan Li, Lexin Wang, Yuanyuan Wang, Lingjun Ma, Ran Zheng, Jingjing Ding, Yichun Gong, Hao Yao, Jue Wang, Xiaoming Zha

**Affiliations:** 1 Department of Breast Disease, The First Affiliated Hospital With Nanjing Medical University, Nanjing, Jiangsu, China

**Keywords:** Breast tumours, Breast surgery, Quality of Life, Patient Reported Outcome Measures, Clinical Trial

## Abstract

**ABSTRACT:**

**Introduction:**

Sentinel lymph node biopsy (SLNB) is a standard procedure for patients with clinically assessed negative axillary lymph nodes (cN0) during early-stage breast cancer (EBC). However, the majority of EBC patients have a negative pathological confirmation of the sentinel lymph node (SLN), and axillary surgery is inevitably associated with postoperative complications. Considering that SLNB has no therapeutic benefit, this trial aims to determine the safety of omitting SLNB in patients with cN0 early invasive breast cancer.

**Methods and analysis:**

The OMSLNB trial is a prospective, single-arm, non-inferiority, phase II, open-label study design involving female breast cancer patients with a tumor of ≤3 cm in diameter, who are considered axillary lymph-node-negative based on two or more radiological examinations, including axillary lymph node ultrasonography. Eligible patients will avoid axillary surgery but will undergo breast surgery, which is not limited to breast-conserving surgery. The trial begins in 2023 and is scheduled to end in 2027. The primary endpoint is 3 year invasive disease-free survival (iDFS). The secondary endpoints include the incidence of breast cancer-related lymphoedema, patient-reported outcomes, locoregional recurrence, local recurrence and regional recurrence. It is expected that the 3 year iDFS in patients undergoing SLNB is about 90%, combined with a non-inferiority cut-off of 5%, 80% power, 95% CIs, 0.05 test level, and 10% loss to follow-up rate, the planned enrollment is 311 patients. All enrolled patients will be included in the intention-to-treat analysis.

**Ethics and dissemination:**

This trial was approved by the Ethics Committee of the First Affiliated Hospital of Nanjing Medical University (No.2023-SR-193). All participants must provide written informed consent to be eligible. The protocol will be described in a peer-reviewed manuscript, and the results will be published in scientific journals and/or at academic conferences.

**Trial registration number:**

NCT05935150.

**Strengths and limitations of this study**

- Patients enrolled in the OMSLNB trial are required to undergo two or more radiological examinations that assess the axillary lymph nodes as negative.
- The OMSLNB study setting up dynamic follow-up at multiple time points, where objective measures will be used to assess the incidence of postoperative arm morbidity and evaluate the quality of life in conjunction with subjective patient-reported outcomes.
- This is a single-centre study, mainly considering that not many hospitals in China are equipped to perform [18F]-fluorodeoxyglucose positron emission/magnetic resonance imaging.

## Introduction

 Since the concept of the sentinel lymph node (SLN) was introduced by Krag *et al* scholars in 1993, axillary surgery for breast cancer progressed towards a minimally invasive era.^1^ The Milan trial, the ALMANAC trial and the NSABP B32 study demonstrated that sentinel lymph node biopsy (SLNB) is a landmark advancement in the field of breast surgery.^2^,^4^ These results confirmed that in patients with negative axillary lymph nodes (cN0) in early-stage breast cancer (EBC), the SLNB group was not significantly different from the axillary lymph node dissection (ALND) group with respect to survival prognosis and local control. However, lymphatic circulation in the upper extremities is more severely impaired in ALND patients, which significantly reduces the quality of life (QoL) rather than contributing a therapeutic benefit.^5^,^8^ Currently, SLNB is recommended as the standard axillary management modality for cN0 patients by several international guidelines.^9^,^11^

SLNB has the potential to result in missed diagnoses as false negative rates (FNR) of 5%–10% are commonly reported.^12^ In the pivotal NSABP B-32 trial,^4^ the FNR for SLNB was 9.8%; however, the 8 year overall survival in the SLNB group was as high as 90.3%, which was not statistically different from that in the ALND group (91.8%) [HR=1.2, 95% CI: 0.96 to 1.50, p=0.12]. In addition, the results of the landmark American College of Surgeons Oncology Group (ACOSOG) Z0011 trial indicated that even in the presence of 1–2 SLNs with macrometastases or micrometastases, performing a more extensive ALND did not improve survival for stage T1-2 patients.^13^ Although non-SLN positivity was detected in 27.3% of the patients in the ALND group, there was no significant difference in the rate of axillary recurrence or DFS in patients in the SLNB group compared with those in the ALND group. This was confirmed in other randomised clinical trials.^14 15^ Other studies reported a low 5 year axillary recurrence rate of 0.3%–2.5% after SLNB,^1316^,^19^ and in a recent prospective SOUND study,^20^ the cumulative incidence of 5 year axillary lymph node recurrence in the omitted SLNB group was only 0.7%. Consequently, these otherwise negative axillary nodes and missed nodes do not appear to pose a serious threat to clinical recurrence and survival. The fact that axillary surgery has never been shown to reduce mortality challenges the premise of the therapeutic efficacy of SLNB.

Although SLNB is a minimally invasive surgical procedure for axillary staging, it is still associated with adverse sequelae. In the ACOSOG Z0011 trial,^13^ approximately one-quarter of the patients in the SLNB group had postoperative complications (including wound infection, lymphedema, axillary sensory abnormalities and brachial plexus injury), and lymphoedema was present in 8% of the patients 6 months after SLNB. The incidence of sensory neuropathy in the SLNB group in the IBCSG 23–01 trial was as high as 13%,^14^ whereas the incidence of lymphoedema was 4%. Similarly, the ALMANAC trial reported a 7% incidence of lymphoedema in the SLNB group.^3^ Considering that SLNB is nontherapeutic and primarily performed for axillary staging, SLNB may represent an overtreatment for SLN-negative patients, and omitting SLNB may result in improved QoL.

In recent years, there have been significant improvements in the accuracy of imaging modalities for preoperative noninvasive axillary staging. A combination of imaging techniques is superior to a single imaging technique for the evaluation of axillary lymph node (ALN) status.^21^ Ultrasonography is the method of choice for ALN assessment in patients with breast cancer.^22^ Choi *et al* found that the specificity of ultrasound assessment of nonpalpable metastatic lymph nodes was 88%–98%.^23^ Another recent meta-analysis revealed that [18F]-fluorodeoxyglucose positron emission/magnetic resonance imaging (FDG PET/MRI) had a higher diagnostic performance compared with MRI in detecting ALN metastases,^24^ but another study found no statistically significant difference between PET/MRI and MRI for N stages.^25^ Preliminary data suggest that PET/MRI has the potential to improve the diagnostic performance of ALN staging, but further studies are needed to confirm and expand the current evidence. Although axillary surgery reduces the risk of axillary recurrence through the local control of the axilla, systemic adjuvant therapy and axillary radiotherapy are also effective. Moreover, postoperative adjuvant therapy should be considered based on the biological characteristics of the tumour rather than the number of ALN involved. Breast cancer treatment decisions frequently rely on tumour biology and genetic profiles. Therefore, the assessment of ALN status is becoming less important.

The OMSLNB trial is designed to evaluate the oncological safety of omitting SLNB for patients with clinically axillary lymph-node-negative, EBC. If the omission of SLNB is noninferior to conventional SLNB for improving survival and controlling recurrence, patients can avoid unnecessary surgery and experience improved QoL with reduced arm morbidity.

Several large prospective clinical trials currently underway, including SOUND,^20^ INSEMA^26^ and the BOOG 2013–08 trial,^27^ are attempting to omit SLNB in patients with cN0 EBC. However, a large number of prospective clinical trials are needed to validate this result, and both of these trials and the OMSLNB trial will provide an important rationale for omitting SLNB in patients with cN0 EBC. This has the potential to change the practice of axillary surgery for EBC in the future.

However, our study possesses several distinct advantages compared with mentioned ongoing trials. First, we have established more stringent inclusion criteria to further enhance the diagnostic performance of ALN staging. Second, given the uncertainty of the tumour location and the actual clinical scenario of a relatively low rate of breast-conserving surgery (BCS) in the Chinese population,^28^ the OMSLNB is the first clinical trial in China to include the omission of axillary surgery in EBC patients planned for mastectomy. Furthermore, this study focuses on women in the Asian Chinese region, where the patient population tends to have a younger age of onset compared with Western patients.

Moreover, the OMSLNB trial will also lay a solid foundation for conducting a future phase III clinical trial. And if the current study achieves its non-inferiority endpoint, we will plan to conduct a larger-scale phase III clinical trial in the future to further validate the efficacy and safety of omitting SLNB.

## Methods and analysis

### Trial design and setting

This study is a prospective, noninferiority, single-arm, phase II open-label clinical trial conducted at the First Hospital of Nanjing Medical University. Patients with unilateral invasive breast cancer (tumor ≤3 cm) and assessed as cN0 will be eligible for inclusion. The enrolled patients are required to complete two or more imaging tests, including axillary ultrasound assessed as axillary lymph-node-negative, and other tests including MRI, PET-CT and [18F]-FDG PET/MRI. Patients with suspected lymph node metastasis will be directly excluded from enrollment. All patients are required to undergo preoperative coarse needle aspiration or intraoperative mass biopsy for pathologically confirmed invasive breast cancer (regardless of pathological type). Subsequently, surgeons will be required to provide a detailed explanation of the potential benefits and risks associated with the omission of SLNB to eligible patients. Before any surgical procedure is undertaken, patients must provide written informed consent. Following consent, patients will undergo BCS or mastectomy (allowing for breast reconstruction) without axillary surgery, with a postoperative follow-up of 3 years.

**Figure 1:**
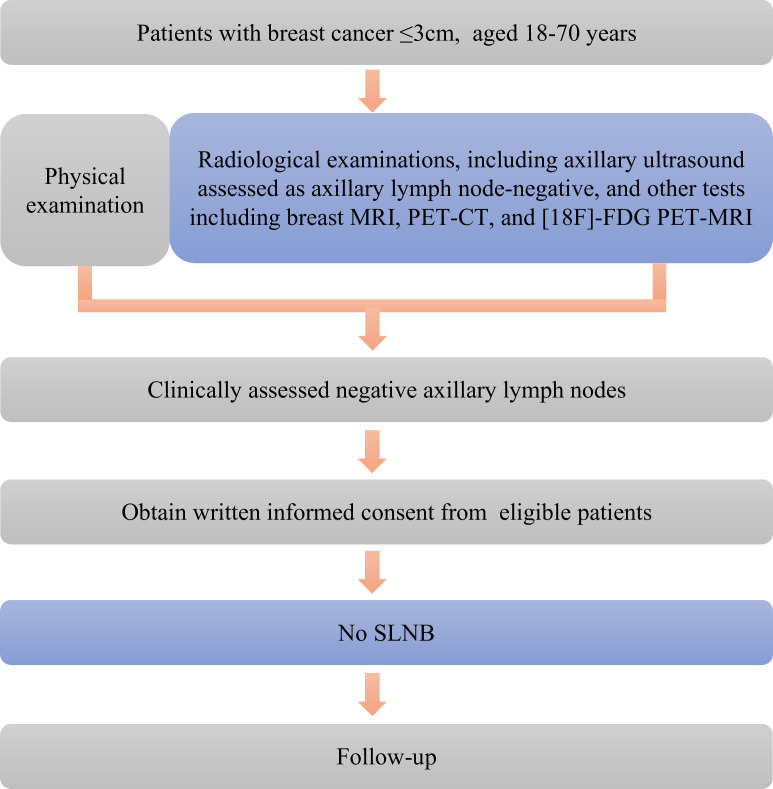
Flow chart of the trial.

This clinical study will be conducted following the relevant provisions of the Declaration of Helsinki, and it was registered at http://www.clinicaltrials.gov (NCT number: NCT05935150). The study was reported according to the Standard Protocol Items: Recommendations for Interventional Trials (SPIRIT) checklist.^29^ The detailed flow chart of the study is shown in figure 1.

### Patient and public involvement

The development of the research question and outcome measures was based on the current clinical context in China and the design of ongoing large-scale prospective clinical studies. Surgeons at the research centre will screen clinical information on patients who are prepared for surgical treatment to assess whether they meet the enrollment criteria. Patients who are willing to participate and eligible for enrollment will then be presented with a detailed explanation of the potential benefits and risks of participating in the study, and written informed consent must be provided for those who agree to enrol. Patients and/or the public were not involved in the design, conduct, reporting or dissemination plans of this research and were not directly involved in the recruitment for this study. We do not plan to proactively disseminate the results to study participants. However, the results will be made available to the general public through publication and other means of dissemination. Participants who are interested in the study results may contact the principal investigator for further information.

### Recruitment

The SPIRIT checklist is presented in table 1. The first participant was enrolled in June 2023. Before the surgery, all planned surgical patients underwent screening of their medical history, physical examination and radiological examination to assess eligibility. For those who were eligible and willing to participate, a detailed explanation of the potential risks and benefits of participating in the study was provided, and all questions were answered. All participants signed a written informed consent form before surgery, one for both the investigator and the participants.

**Table 1:** The schedule of enrolment, interventions and assessments based on the SPIRIT checklist.

Timepoint	Study period									
	Screening and enrolment	Trial treatment period	Follow-up period							
	−1 w	0 (Operation)	1w	1m	3m	6m	1y	1.5y	2y	3y
Enrollment										
Collection of informed consent	X									
Demographic and clinicopathological data	X									
Medical history	X									
Physical examination*	X				X	X	X	X	X	X
Radiological examination†	X				X	X	X	X	X	X
Histological or cytological confirmation of breast cancer	X									
Interventions										
Omit SLNB‡		X								
Breast surgery		X								
Adjuvant systemic therapy (if implemented)		X								
Radiotherapy (if implemented)		X								
Assessments										
Arm circumference measures			X	X	X	X	X	X	X	X
Bioelectrical impedance			X	X	X	X	X	X	X	X
Questionnaire Quick DASH			X	X	X	X	X	X	X	X
Questionnaire FACT B			X	X	X	X	X	X	X	X
Survival outcome			X	X	X	X	X	X	X	X
Disease status			X	X	X	X	X	X	X	X
Adverse events			X	X	X	X	X	X	X	X

* Physical examination, including breast and axillary lymph node palpation.

† Radiological examinations, including breast and axillary ultrasound, MRI, PET-CT, and [18F]-FDG PET-MRI inspection projects.

‡ SLNB, sentinel lymph node biopsy.

### Trial participants and inclusion/exclusion criteria

#### Inclusion criteria

The study enrolled participants based on strict inclusion and exclusion criteria. Patients who meet all of the following inclusion criteria will be eligible for inclusion:

1. Female patients aged 18–70 years.
2. Pathologically confirmed invasive breast cancer (regardless of he pathological type), breast cancer of ≤3 cm and scheduled to undergo breast surgery.
3. Negative axillary lymph nodes as assessed by physical examination and two or more radiological exams, including axillary ultrasound, such as breast MRI, breast PET-CT or breast PET/MRI.
4. All patients are required to undergo immunohistochemical staining for oestrogen receptor, progesterone receptor, human epidermal growth factor receptor 2 (HER2), Ki-67 proliferation index and further fluorescence in situ hybridisation should be performed in HER2 2+cases.
5. Good compliance, normal comprehension and the ability to receive treatment and follow-up as required.
6. ECOG score 0–1.
7. Patients who volunteered for this study signed the informed consent form.

#### Exclusion criteria

Patients meeting any of the following exclusion criteria will be excluded from the study:

1. Bilateral, lactating, pregnant breast cancer.
2. Clinical or imaging confirmation of distant metastasis.
3. Previous history of malignant tumour or neoplasm.
4. Previous surgery on the affected axilla or history of surgery affecting the function of the upper extremity.
5. History of radiotherapy to the breast or chest.
6. Positive pathological margins after BCS or mastectomy.
7. Severe coagulation disorder, serious systemic disease or an uncontrollable infection.
8. Aspartate aminotransferase (AST), alanine aminotransferase (ALT) of ≥1.5-fold of the upper limit of normal value, alkaline phosphatase of ≥2.5-fold of the upper limit, total bilirubin of ≥1.5-fold of the upper limit, serum creatinine of ≥1.5-fold the upper limit and left ventricular injection fraction (LVEF) of <50% on cardiac ultrasound.
9. Inability to complete the full course of follow-up adjuvant therapy as prescribed by the doctor for various reasons.
10. No personal freedom or independent civil capacity.
11. Presence of mental disorders, addictions, etc.
12. Not eligible for enrollment as judged by the investigator.

### Clinical intervention and follow-up

After obtaining informed consent, axillary surgery will be omitted for eligible patients, and BCS or mastectomy (allowing breast reconstruction) will be voluntarily selected by the patient under the premise of ensuring therapeutic efficacy. Patients receiving BCS must undergo whole breast irradiation (WBI) after surgery, and postoperative adjuvant therapy and other treatments will be performed according to clinical guidelines.

Multiple timepoints will be established for dynamic follow-up at baseline and up to 3 years postoperatively, with a comprehensive and objective assessment of breast cancer-related lymphoedema (BCRL) using multi-segmental arm circumference measurements and bioelectrical impedance analysis at baseline, 1 week, 1, 3, 6, 12, 18, 24, and 36 months postoperatively, respectively. Patients will be administered a QoL questionnaire using the validated FACT-B (Version 4.0) and Quick DASH at the same time point.^30 31^ BCRL incidence and QoL will be subjectively assessed by patients using patient-reported outcomes (PROs).

Patients who discontinue or withdraw from the trial for any reason during the trial can be treated with disease-specific therapies, including supplemental SLNB, ALND, radiotherapy and other therapies as appropriate, after obtaining informed consent.

### Postoperative radiotherapy

Adjuvant WBI with 3D conformal radiation therapy or intensity-modulated radiation therapy to the remaining breast is mandatory after BCS. The radiotherapy target area will not include the axillary region, and chest wall irradiation can be omitted with mastectomy. In this trial, either conventional fractionated radiotherapy or macrofractionated radiotherapy regimens will be used. The conventional fractionated whole breast radiotherapy dose will be 50 Grey (Gy) in 25 fractions, with a single dose of 2.0 Gy, five times per week. The tumour bed area will be selected to be added simultaneously or sequentially to DT60 Gy in 25 fractions, with a single dose of 2.4 Gy in fraction. The macro-fractionated radiotherapy whole breast radiotherapy dose will be 43.5 Gy in 15 fractions with a single dose of 2.9 Gy. The tumour bed area will be increased to 52.5 Gy in 18 fractions with a single dose of 2.9 Gy.

### Adjuvant systemic treatment

Systemic adjuvant therapies will be administered in accordance with local and international guidelines, including adjuvant chemotherapy, targeted therapy, endocrine therapy and immunotherapy are administered in accordance with nationally approved product information. Postoperative adjuvant therapy is required for HER2-positive or triple-negative breast cancer, regardless of ALN status. For HR+/HER2–, genetic testing may be used to guide decisions regarding adjuvant chemotherapy.

### Endpoints

#### Primary endpoint

The primary endpoint of this trial is 3 year invasive disease-free survival (iDFS), defined as the time from surgery to local recurrence, regional recurrence, distant metastasis, infiltration of the contralateral breast cancer or death from any cause, whichever occurs first.

#### Secondary endpoints

- BCRL incidence: two measurements will be used to diagnose BCRL, and BCRL is diagnosed by meeting one of them.

##### 1. Relative volume change (RVC) of >10% in the affected upper limb

The upper limb volume will be calculated using the method of multisegmental arm circumference measurement. Five points will be taken to measure arm circumference. The midpoint of the ulnar styloid process at the distal end of the arm will be the starting point and measurements will be taken every 10 cm from this point towards the proximal end of the arm up to 40 cm. These five measurement points enable the limb to be divided into four truncated cones. The volume of each limb segment is calculated using the following formula:

V=[h (C12+C22+C1C2)]/12π

where C1 and C2 denote the arm circumference at the upper and lower points of the measurement segment, h=10 cm. The volume of the entire upper limb is the sum of the volumes of the segments. RVC is calculated using the following formula^32^:

RVC=[(A2×U1])/(U2×A1)]−1

where A1 indicates the volume of the affected upper limb at baseline, A2 indicates the volume of the affected upper limb at a specific time point, U1 represents the volume of the unaffected upper limb at baseline and U2 indicates the volume of the unaffected upper limb at a specific time point. The incidence of BCRL will be assessed at each follow-up timepoint.

##### 2. Bioelectrical impedance analysis (BIA)

The single-frequency bioimpedance analysis (SFBIA) ratio is defined as the ratio of the unaffected to the affected side.^33^ The SFBIA ratio of both upper limbs at 1 kHz and 5 kHz will be measured using bioelectrical impedance technology. The diagnostic criteria in this study will refer to the findings of Liu Miao *et al*. BCRL recognition is superior when the threshold is set at the mean+2 SD for healthy women.^34^ BCRL will be diagnosed when any one of the following is fulfilled:

1. The dominant affected arm SFBIA ratio at 1 kHz of >1.067.
2. The nondominant affected arm SFBIA ratio at 1 kHz of >1.043.
3. The dominant affected arm SFBIA ratio at 5 kHz of >1.068.
4. The nondominant affected arm SFBIA ratio at 5 kHz of >1.044.
  - PROs: Patients will be followed up with questionnaires using FACT-B (Version 4.0) and Quick DASH during the same period as the BCRL assessment. The FACT-B scale will be used to assess changes in QoL in breast cancer patients with SLNB omission from baseline to 3 years after surgery. The Quick DASH scale will be used to assess changes in arm morbidity from baseline to 3 years after surgery.
  - Locoregional recurrence (LRR), local recurrence (LR), regional recurrence (RR)： LR is defined as the time from surgery to the appearance of a tumour in the ipsilateral breast, chest wall or skin at the surgical scar. RR is defined as the time from surgery to the appearance of a tumour in the lymphatic drainage area of the affected side, including the axillary, supraclavicular, subclavicular and internal mammary lymph node regions. Clinical recurrence suggested by imaging or other investigations requires a definitive diagnosis by cytology or histopathology. Suspected recurrent lesions for which histological or cytological examination is not possible will be submitted to the Data Safety Monitoring Board (DSMB) for independent review.

### Sample size calculation

In this study, we used PASS software (version 2021) to perform a sample size calculation for a one-proportion non-inferiority test. The sample size was based on the primary endpoint of iDFS. According to the literature exploration and previous research results, it is expected that the 3 year iDFS in patients undergoing SLNB is about 90%. We set the non-inferiority margin at 5%, with a power of 80%, a significance level (α) of 0.05 and a 95% CI. Considering a 10% dropout rate, PASS software calculated the required sample size to be 311 patients.

### Data collection and management

This study uses an electronic case report form to manage and record the study data, and a password-protected access system will be set up to maintain participant confidentiality. The principal investigator has full responsibility of entire projects of this trial. The researchers needed to ensure that the data entry and correction were timely, complete, accurate and truthful. To ensure the accuracy of the data, double-entry and proofreading will be done independently by two data administrators. The clinical supervisor will perform data checking after the end of the study. The researcher will reply and follow-up on corrections promptly when the data are found to be incomplete inconsistent are challenged. If a subject withdraws their informed consent during participation in a clinical trial, no data will be collected after withdrawal. Study-related data collected before withdrawal will be retained and used for up to 3 years after withdrawal. At the end of the study, the principal investigator will submit a final report for review and written approval by the ethics committee.

### Statistical analysis

The primary analysis will use an intention-to-treat (ITT) analysis. For categorical variables, the χ2 test or Fisher’ s exact test will be used, and the data will be presented as totals, percentiles and frequencies. For continuous variables, descriptive statistics will be analysed using mean, SD or median and IQR. Independent samples t-tests will be used for normally distributed continuous variables, and nonparametric tests will be used for skewed data to analyse the results. Kaplan–Meier curves and log-rank tests will be used to determine the statistical significance of variables in the survival outcome categories (iDFS) and survival curves will be plotted. The prognostic significance of variables will be assessed using univariate and multivariate COX risk regression analyses. HR and 95% CI will be calculated. For each statistical analysis, a p value <0.05 will be considered statistically significant.

### Missing data and dropouts

Patients who discontinue or withdraw from the trial for any reason during the trial can be treated with disease-specific therapies and will not adversely affect their subsequent treatment, including supplemental SLNB, ALND, radiotherapy and other therapies as appropriate, after obtaining informed consent. For missing data, we will not perform any imputation. Instead, we will use the last observation carried forward method, which helps to maintain the integrity of the ITT analysis.

### Data monitoring

An independent DSMB will be established for this study and will be reviewed every 6 months. The Data Supervision Committee will be responsible for the supervision and management of the research process and the authenticity of the data. The Safety Monitoring Board will be responsible for monitoring and analysing the adverse events (AEs) of the trial and guiding the management of AEs in accordance with the operational procedures and clinical principles. Any serious adverse events (SAEs) that occur during the course of this clinical trial will follow the National Cancer Institute’s Criteria for Common Toxic Reactions (NCI/CTCAE v5.0). The investigator will report in writing all SAEs, suspected and unexpected SAEs, and unexpected events that occur during the trial to the sponsor and the ethics committee within 24 hours of becoming aware of them. This will ensure that patients are treated promptly and documented in detail. Interim analyses will be performed by a statistician and reported to the DSMB, which will have access to all data and will discuss the results of interim analyses with the principal investigator and report to the ethics committee, which will ultimately decide whether the study should proceed.

### Ethics and dissemination

The study will be conducted in strict accordance with the tenets of the Declaration of Helsinki, and all patient data will be processed anonymously. The protocol was approved by the Ethics Committee of the First Affiliated Hospital of Nanjing Medical University on 16 May 2023 (Len review number: 2023 SR-193). Any amendments to the approved clinical trial protocol, informed consent and other study materials will be reviewed by the Ethics Committee of the First Affiliated Hospital of Nanjing Medical University and implemented after approval. The results of this study will be disseminated through academic conferences and peer-reviewed journals.

### Trial status

The protocol version number and date were V3.0 and 21 May 2023. The study was conceived and designed in 2022. The first patient was enrolled on 28 June 2023, and enrolment is expected to be completed in June 2026. At the time of manuscript submission, the study was in the process of enrolling patients.

### Consent or assent

Before any surgical procedures, researchers will provide a detailed explanation of the potential benefits and risks associated with the omission of SLNB to eligible and consenting participants. A dedicated period will be reserved to address all questions raised by the participants. Following this, the investigators will request that the participants sign an informed consent form. Participants have the right to withdraw from the study at any time and for any reason, without prejudice. Should a participant choose to withdraw completely, their data will not be included in the final data analysis.
