# Supplementary material for: Omission of sentinel lymph node biopsy in patients with clinically axillary lymph node-negative early breast cancer (OMSLNB): protocol for a prospective, non-inferiority, single-arm, phase II clinical trial in China
Source: BMJ Open. 2024 Sep 10;14(9):e087700. doi: 10.1136/bmjopen-2024-087700 (PMC11409317; doi:10.1136/bmjopen-2024-087700)
Supplement: Supplementary file 1 [file bmjopen-2024-087700.reviewer_comments.pdf]

PEER REVIEW HISTORY

BMJ Open publishes all reviews undertaken for accepted manuscripts. Reviewers are asked to complete a checklist review form and are provided with free text boxes to elaborate on their assessment. These free text comments are reproduced below.

ARTICLE DETAILS

**TITLE (PROVISIONAL)**	Omission of sentinel lymph node biopsy in patients with clinically axillary lymph node-negative early breast cancer (OMSLNB): protocol for a prospective, noninferiority, single-arm, phase II clinical trial in China
**AUTHORS**	Li, Xuan; Wang, Lexin; Wang, Yuanyuan; Ma, Lingjun; Zheng, Ran; Ding, Jingjing; Gong, Yichun; Yao, Hao; Zha, Xiaoming; Wang, Jue

VERSION 1 - REVIEW

**REVIEWER NAME**	***Hiller, Louise***
**REVIEWER AFFILIATION**	University of Warwick
**REVIEWER CONFLICT OF INTEREST**	Na
**DATE REVIEW RETURNED**	28-May-2024

**GENERAL COMMENTS**	The study is well designed, and the protocol is clearly written and logically presented. My only comments are:<br>- The text is very clear that “The enrolled patients are required to complete two or more imaging tests, including axillary ultrasound assessed as axillary lymph node-negative, and other tests including MRI, PET-CT, and [18F]-FDG PET-MRI.” However, this is not reflected in Figure 1, where it appears that, after informed consent, patients either have a physical exam or a radiological assessment…?<br><br>There is no explanation about whether this is an ITT or PPA. i.e. Are they are going to exclude from analysis patients who subsequently refuse RT?<br><br>It doesn't say anywhere if findings will lead into a larger phase III confirmatory trial.<br><br>Sample size paragraph requires further information to allow for replication of numbers e.g. which package was used.

**REVIEWER NAME**	***Damin, Andrea***
**REVIEWER AFFILIATION**	Hospital de Clinicas de Porto Alegre, Breast Surgery
**REVIEWER CONFLICT OF INTEREST**	Na
**DATE REVIEW RETURNED**	02-Jul-2024

**GENERAL COMMENTS**	The project addresses a very interesting topic. The omission of sentinel node biopsy is still subject in discussion. This trial maybe can add important information<br>There are minor revisions that need to be made in the Spirit check

	list<br>1 . No informed consent was provided, only mention on the text<br>2. in consent or assent topic: they need explain who will obtain informed consent<br>3. statistic outcome were in page 16 not 15

VERSION 1 – AUTHOR RESPONSE

Response to Reviewer# 1's comments :

Comment 1:
The text is very clear that "The enrolled patients are required to complete two or more imaging tests, including axillary ultrasound assessed as axillary lymph node-negative, and other tests including MRI, PET-CT, and [18F]-FDG PET-MRI." However, this is not reflected in Figure 1, where it appears that, after informed consent, patients either have a physical exam or a radiological assessment…?

Response:
Thank you for highlighting the inconsistency between the text and Figure 1. We have revised Figure 1 (page 6) to reflect the correct sequence of events. Specifically, written informed consent should be obtained for eligible patients after completing physical and imaging assessments. The revised figure now accurately represents the workflow as described in the text. Figure 1 is as follows:

Comment 2:
There is no explanation about whether this is an ITT or PPA. i.e., Are they going to exclude from analysis patients who subsequently refuse RT?

Response:
We appreciate your attention to the analytical approach. Our study employs an intention-to-treat (ITT) analysis. As a single-arm clinical trial, the ITT population includes all participants who signed the informed consent form and met the inclusion and exclusion criteria. This method ensures that all participants are analyzed, regardless of whether they completed the intervention or follow-up as planned. We have added a clarification in the "Methods and Analysis" section on lines 36-37 of page 1 and in the "Statistical Analysis" section on lines 4-5 of page 11 of the manuscrip to explicitly state this approach. The changes are as follows:
All enrolled patients will be included in the intention-to-treat analysis. (page 1, lines 36-37)
The primary analysis will use an intention-to-treat (ITT) analysis. (Page 11, lines 4-5)
And as you have given the example, patients who subsequently refuse RT will also be included in the final analysis.

Comment 3:
It doesn't say anywhere if findings will lead into a larger phase III confirmatory trial.

Response:
Thank you for raising this important point. We agree that it is crucial to outline the potential future steps based on the findings of this study, if the current study achieves its non-inferiority endpoint, we will plan to conduct a larger-scale phase III clinical trial in the future to further validate the efficacy and safety of omitting SLNB.
And we have already provided a supplementary explanation in the Introduction section at the end of the article on page 4, lines26-30. The modification is as follows:
Moreover, the OMSLNB tria will also lay a solid foundation for conducting a future phase III clinical trial. And if the current study achieves its non-inferiority endpoint, we will plan to conduct a larger-scale phase III clinical trial in the future to further validate the efficacy and safety of omitting SLNB.

Comment 4:
Sample size paragraph requires further information to allow for replication of numbers e.g. which package was used.

Response:
Thank you for your significant reminding. We have provided additional detail regarding the sample size calculation. Our study is a prospective, non-inferiority, single-arm clinical trial. Therefore, we used PASS software to perform a sample size calculation for a single proportion non-inferiority test. We have included a detailed description of the sample size calculation in the "Sample size calculation" section on page 10, lines 34-41 of the manuscript. The corresponding changes are as shown below:
In this study, we used PASS Software (version 2021) to perform a sample size calculation for a one-proportion non-inferiority test. The sample size was based on the primary endpoint of iDFS. According to the literature exploration and previous research results, it is expected that the 3-year iDFS in patients undergoing SLNB is about 90%. We set the non-inferiority margin at 5%, with a power of 80%, a significance level (α) of 0.05, and a 95% confidence interval. Considering a 10% dropout rate, PASS software calculated the required sample size to be 311 patients.

Response to Reviewer #2's comments :

Comment 1:
No informed consent was provided, only mention on the text.

Response:
We apologize for the oversight. We have uploaded the Patient Informed Consent form as supplemental material. Since our study targets a Chinese population, the consent form is written in Chinese. To facilitate reading by other readers, we have included an English version following the Chinese version.

Comment 2:
In consent or assent topic: they need to explain who will obtain informed consent.

Response:
Thank you for this suggestion. We have revised the manuscript to clarify that written informed consent must be obtained from the patients. Specifically, we have updated the "Consent or assent " section on lines 3-10 of page 12 to include this information. The details of the modification are as follows:
Consent or assent
Prior to any surgical procedures, researchers will provide a detailed explanation of the potential benefits and risks associated with the omission of SLNB to eligible and consenting participants. A dedicated period will be reserved to address all questions raised by the participants. Following this, the investigators will request that the participants sign an informed consent form. Participants have the right to withdraw from the study at any time and for any reason, without prejudice. Should a participant choose to withdraw completely, their data will not be included in the final data analysis.
Additionally, we have detailed the consent process in the "Trial Design and Setting" section on page 5, lines 15-20 , emphasizing that: surgeons will be required to provide a detailed explanation of the potential benefits and risks associated with the omission of SLNB to eligible patients. Before any surgical procedure is undertaken, patients must provide written informed consent. Following consent, patients will undergo BCS or mastectomy (allowing for breast reconstruction) without axillary surgery.

Comment 3:
Statistic outcome were in page 16 not 15.

Response:
Thank you for identifying this issue. We have carefully reviewed and updated the page numbers in the SPIRIT checklist to ensure accuracy and consistency with the manuscript. And in the revised manuscript, statistic outcome is on page 11.
